# The impact of control strategies and behavioural changes on the elimination of Ebola from Lofa County, Liberia

**DOI:** 10.1098/rstb.2016.0302

**Published:** 2017-04-10

**Authors:** Sebastian Funk, Iza Ciglenecki, Amanda Tiffany, Etienne Gignoux, Anton Camacho, Rosalind M. Eggo, Adam J. Kucharski, W. John Edmunds, Josephus Bolongei, Phillip Azuma, Peter Clement, Tamba S. Alpha, Esther Sterk, Barbara Telfer, Gregory Engel, Lucy Anne Parker, Motoi Suzuki, Nico Heijenberg, Bruce Reeder

**Affiliations:** 1 Centre for the Mathematical Modelling of Infectious Diseases, London School of Hygiene and Tropical Medicine, London WC1E, UK; 2 Operational Centre Geneva, Médecins Sans Frontières, 1211 Geneva, Switzerland; 3 Epicentre, 1211 Geneva, Switzerland; 4 Lofa County Health Office, Ministry of Health and Social Welfare, 7500 Voinjama, Liberia; 5 Monrovia Country Office, World Health Organization, 1000 Monrovia, Liberia; 6 CIBER Epidemiología y Salud Pública, 28029 Madrid, Spain; 7 Department of Public Health, Universidad Miguel Hernández, 03202 Alicante, Spain; 8 Institute of Tropical Medicine, Nagasaki University, Nagasaki, Japan; 9 College of Medicine, University of Saskatchewan, Saskatoon, Canada S7N 5E5

**Keywords:** infectious disease dynamics, mathematical model, behavioural changes, interventions, community engagement, isolation

## Abstract

The Ebola epidemic in West Africa was stopped by an enormous concerted effort of local communities and national and international organizations. It is not clear, however, how much the public health response and behavioural changes in affected communities, respectively, contributed to ending the outbreak. Here, we analyse the epidemic in Lofa County, Liberia, lasting from March to November 2014, by reporting a comprehensive time line of events and estimating the time-varying transmission intensity using a mathematical model of Ebola transmission. Model fits to the epidemic show an alternation of peaks and troughs in transmission, consistent with highly heterogeneous spread. This is combined with an overall decline in the reproduction number of Ebola transmission from early August, coinciding with an expansion of the local Ebola treatment centre. We estimate that healthcare seeking approximately doubled over the course of the outbreak, and that isolation of those seeking healthcare reduced their reproduction number by 62% (mean estimate, 95% credible interval (CI) 59–66). Both expansion of bed availability and improved healthcare seeking contributed to ending the epidemic, highlighting the importance of community engagement alongside clinical intervention.

This article is part of the themed issue ‘The 2013–2016 West African Ebola epidemic: data, decision-making and disease control’.

## 1. Introduction

The epidemic of Ebola virus disease (EVD) in West Africa, has, at the time of writing, caused more than 11 000 deaths reported from more than 28 000 cases [1]. The outbreak was first identified in March 2014 in South Western Guinea [2–4]. By 30 March 2014, the infection had spread across the border, and the first cases were reported from Liberia, more specifically from Lofa County near the northern border with Guinea. Following this first small cluster of cases, in early June, a large outbreak was observed in Lofa, with rapid growth in case numbers between early July and late August. The outbreak peaked in August before declining in September and decreasing to zero cases of EVD by early November [5,6].

Ebola spreads through contact with bodily fluids that continue to be infectious after death [7]. This makes finding and isolating infected patients and preventing transmission at funerals crucial components of the public health response [8]. Ebola treatment centres (ETC) are specifically designed to provide isolation beds for infected patients, with staff trained to minimize the risk of onward transmission to themselves or others by wearing protective gear and practising the highest levels of hygiene. Infection at funerals can be prevented by conducting burials where only those wearing appropriate equipment touch the body of the deceased.

The exponential rise in case numbers in the summer of 2014 in Lofa County and elsewhere triggered a large-scale international response of humanitarian assistance to West Africa. In Lofa County, this led to a rapid expansion in ETC bed capacity, from 10 beds in June 2014 to about 100 by mid-August. Beyond its humanitarian contribution through the provision of appropriate care for a greater number of patients, providing a sufficient number of beds can contribute to curbing an epidemic by removing cases from the community and isolating them, thereby preventing further spread.

As much of transmission of Ebola occurs in (not necessarily nuclear) families, communities and at funerals, behaviour change can have a great impact on the course of an outbreak. This makes it important to understand the cultural contexts and processes that can trigger such change as well as its impact on the transmission [9]. Experiences in earlier outbreaks of Marburg virus disease in Angola [10] and EVD in Uganda [11] and in Sierra Leone during the current outbreak [12], emphasize the importance of health promotion interventions, in particular the need to understand the local context and culture, and work closely with community leaders to adapt the intervention to the setting [13,14]. World Health Organization (WHO) Standard Operating Procedures for controlling Ebola and Marburg virus epidemics highlight the importance of a comprehensive, integrated approach [15]. Around the same time as the expansion of ETC bed capacity, a suite of community-based health promotion and social mobilization activities were launched in Lofa County. To what extent these activities and ETC bed expansion contributed to end the epidemic is unclear.

Mathematical modelling of EVD epidemics has previously provided insights into the different routes of Ebola transmission [16,17] and the potential role of different control strategies, including interventions based on case management and safe handling of dead bodies as well as community-based interventions [18–23]. A recent modelling study has suggested that the spread of awareness and consequent self-initiated behavioural changes may have played a substantial role in turning the epidemic in Lofa County [24], but did not include a full account of the public health response. Another study has suggested that community transmission of Ebola in Liberia was reduced by 40–80% over the course of the outbreak. The provision of hospital beds, on the other hand, has been estimated to have averted more than 50 000 cases in Sierra Leone [25]. A recent study has found that control of the Ebola epidemic in Liberia was achieved through reductions in community transmission [26].

Here, we aim to characterize the epidemiological features of, and the public health response to, the EVD outbreak in Lofa County in 2014, and assess the role of the multiple components of intervention in the control of the outbreak. To tease out the impact of different clinical and community-based interventions, and to estimate the relative contribution of these to containing the epidemic in Lofa County, we fitted a mathematical model of Ebola virus transmission with a time-varying transmission rate to the series of ETC admissions. This allowed us to fully account for the progression of EVD in infected individuals and associated delays, and thus to pinpoint when declines or increases in transmission rates must have occurred to produce the observed time series of cases.

## 2. Methods

### (a) Data

To record suspect cases of EVD and suspect community deaths, a case notification form was completed by the district health officer (DHO) or member of their staff. This identified: symptoms, date of onset of symptoms, prior exposure to a known case of EVD, attendance at a funeral or traditional healer, and family or community contacts to whom the individual may have transmitted the infection. Cases were considered suspect, probable or confirmed according to WHO and Liberian Ministry of Health and Social Welfare (MoH) case definitions [27]. Laboratory examination was conducted whenever possible to confirm the diagnosis: reverse transcriptase-polymerase chain reaction (RT-PCR) analysis was performed on a sample of whole venous blood from patients admitted to the ETC or on an oral mucosal swab from community deaths by the Institut Pasteur laboratory, Conakry, Guinea (30 March–4 April 2014), the European Mobile Laboratory based in Guéckédou, Guinea (4 April–12 September 2014) and later the European Mobile Laboratory in Foya, Liberia (13 September–10 November 2014) [28,29]. From the line lists of suspect, probable and confirmed cases of EVD in the ETC and reported community deaths, we extracted the time course of ETC admissions and distribution of times from onset to death, and for ETC cases the distributions of time from the onset of symptoms to admission and length of stay. For calculating admission delays, we removed two patients with reported delays of more than three weeks, and for calculating the time from onset to deaths, two patients with reported times of more than a month.

During the outbreak in Lofa County, a complete registry of deaths was not maintained and formal population-based surveys of knowledge, attitudes and behaviour relevant to EVD were not conducted. The chronology and nature of the interventions of various actors were derived from operational records, through participant observation and unstructured, qualitative interviews with key informants in participating organizations and the community. Media messages in the county were also informally monitored. Community-based interventions to control the EVD outbreak included: social mobilization to influence community knowledge, attitudes and behaviour; and outreach teams that assessed and transported suspect cases, provided safe burials and disinfected dwellings. The MoH, Médecins Sans Frontières (MSF) and UNICEF led the former activity, whereas the MoH and MSF provided the latter services. The number of individuals contacted per week by the MSF health promotion teams is presented as a measure of these community-based activities.

### (b) Transmission model

**Figure 1.**
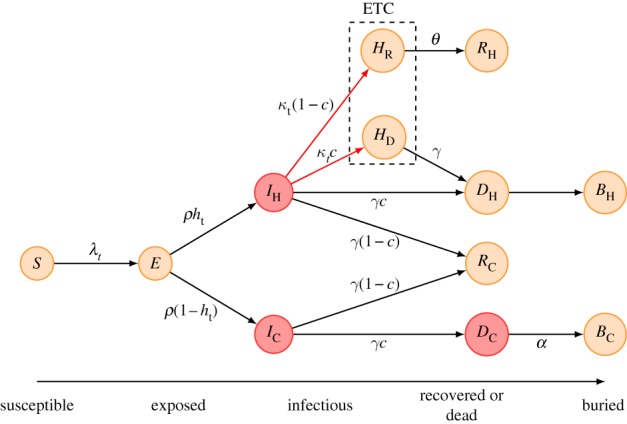
Schematic of the model. The transitions used to calculate the weekly incidence of ETU admissions that was compared with the data are coloured in red, infectious compartments are shaded red and the compartments inside the ETC marked by a box.

A stochastic compartmental model of Ebola virus transmission was constructed and parametrized using detailed information obtained from the line list of ETC admissions. The model extended the classic susceptible–exposed–infectious–recovered (SEIR) model as follows: at time *t*, susceptibles (*S*) experience a (time-varying) force of infection *λ*(*t*). Upon infection, susceptibles move into the exposed (*E*) compartment, representing those infected but not yet infectious. After the incubation period, people in the exposed class move into one of two infectious (*I*) classes (with rate *ρ*), depending on whether they are healthcare seeking (*I*_H_, for (H)ospital, where these individuals may ultimately arrive) or not (*I*_C_, for (C)ommunity, where these individuals will remain). With time-varying probability *h*_t_, individuals leaving the *E* compartment enter the *I*_H_ compartment where they remain in the community until they get admitted to the ETC (*H*_R_ or *H*_D_, with time-varying rate *κ*_t_), recover (*R*_C_) or die (*D*_H_, both with rate *γ*) and get a safe burial (*B*_H_). With (time-varying) probability (1 − *h*_t_), they enter the *I*_C_ compartment where they remain until they recover (*R*_C_) or die (*D*_C_, both with rate *γ*) and get an unsafe burial (*B*_C_, at rate *α*). Individuals from the *I*_H_ compartment who get admitted to the ETC are split into those (*H*_D_) who eventually die (*D*_H_) and get a safe burial, and those (*H*_R_) who recover (*R*_H_), with the relative probabilities of entering the *H*_D_ or *H*_R_ compartments given by the case fatality rate *c*. Hospital admissions (into the *H*_R_ and *H*_D_ compartments) occur with multiplicative gamma-distributed noise (mean 1, standard deviation 0.1), a convenient choice for a rate that is subject to random variation [30,31]. A schematic illustration of the model is given in figure 1.

In the model, when infectious people are split into those that seek healthcare (*I*_H_) and those that do not (*I*_C_), the ones that would seek healthcare (i.e. are in *I*_H_) but do not get to the ETC (because they die or recover before) are safely buried. The ones who would not seek healthcare (i.e. are in *I*_C_) are not safely buried and remain infectious (in *D*_C_) until they are buried (*B*_C_). While we did not have any data to inform this choice, the rationale is that these might be part of resistant communities who would not call for a safe burial. Note that, as healthcare seeking changes during the course of the outbreak, so does the proportion of dead that is safely buried.

Because we had reports that no patient arriving at the ETC had ever been rejected in Lofa, we did not include any effects of ETC capacity and occupancy in fitting the model to the epidemic trajectory. When considering counterfactual scenarios later, however, we did include one where ETC capacity was limited and patients rejected if it was full (see section ‘Counterfactual scenarios’ below).

Because a focus of this work was on timing, we used the patient data to parametrize the model with realistic transition time distributions. Transition times in standard compartmental models can be made to follow gamma distributions with integer shape parameters, that is Erlang distributions, by putting compartments in sequence [32]. We fitted gamma distributions to the observed distributions of the admission delay, the length of stay of survivors and the time from onset to death of casualties, with rounded-down and rounded-up shape parameters using maximum-likelihood estimation with the *R* [33] package *fitdistrplus* [34], and chose the one with the greater likelihood as an estimate of the shape parameter of the Erlang distribution corresponding to each transition. The compartments were then split into the number of subcompartments given by the shape parameter, and transition rate multiplied with the same shape parameter, to recover the correct mean transition times. Where individuals were subject to multiple Erlang-distributed transitions (in *I*_H_), they would have an index for each of these transitions. The index with respect to any transition was maintained under other transitions such that, for example, people admitted to the ETC late in their infectious period would proceed to death quicker than those who were admitted early in the infectious period.

We assumed that the symptomatic period coincided with the infectious period, as suggested by a study of a previous Ebola outbreak where infection was found to have been transmitted by ill patients both in early and late stages of illness [35]. We further assumed that infectiousness was the same for those who proceeded to recovery or death, and did not change after death until buried (unless safely buried), the simplest assumption in the absence of any data to inform this matter. We did, however, consider a modified model where transmissibility increased late in the infectious period and in an unsafe funeral when calculating the effect of isolation on the reproduction number, as it has been suggested that patients might become more infectious during later stages of their infection [19,35].

**Figure 2.**
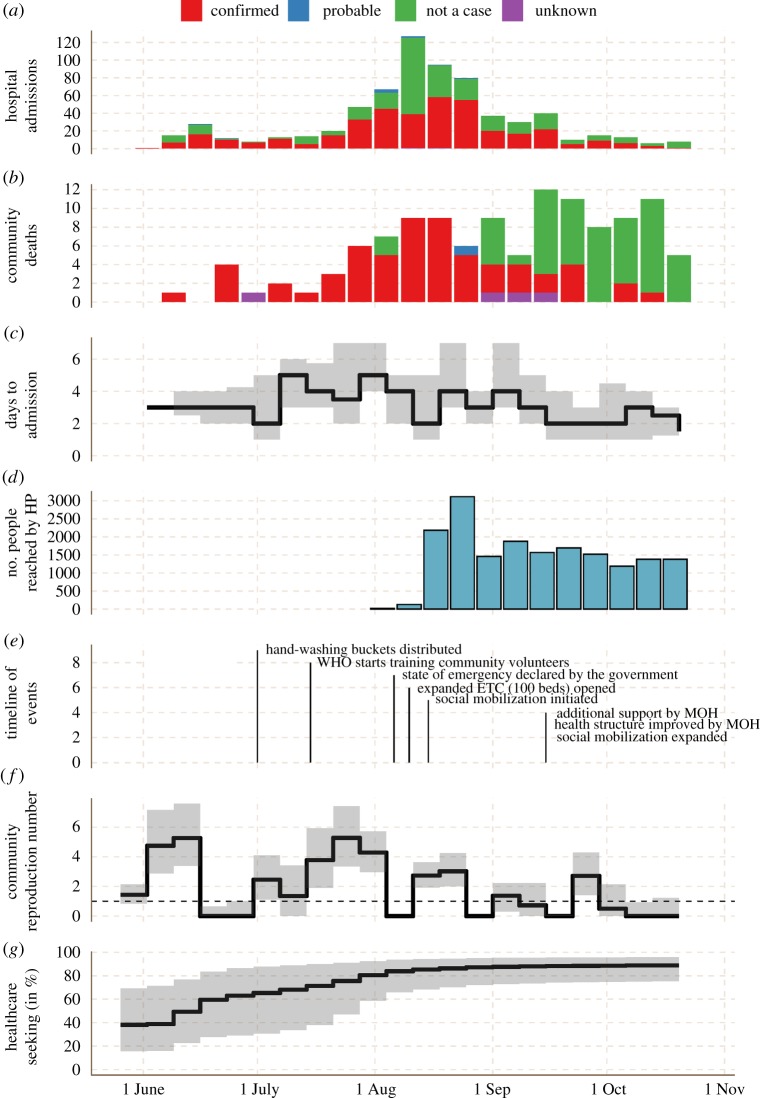
Timeline of the EVD epidemic in Lofa County. (*a*–*e*) Data: number of hospital admissions, community deaths, days from onset to ETC admission (median and IQR), people reached by MSF health promotion (HP) activities, time line of events. (*f*,*g*) Modelling results: community reproduction number (i.e. the reproduction number in the absence of isolation beds) and the proportion of patients seeking healthcare. Black line: median; grey area: interquartile range.

We included the time-varying admission delay by weekly varying the rate of admission *κ*_t_ according to the observed mean (figure 2). The case fatality rate was extracted from the line list as 67%, and the population size of Lofa County set to *N* = 270 114 [36]. The incubation period was set to 9.4 days with shape parameter 2 [37]. The average period of infectiousness after death for those unsafely buried was taken to be 1 day [17]. All delays and their corresponding parameters are summarized in table 1.

**Table 1.** Delays in the model, the corresponding rate parameter, the mean of the delay (the reciprocal of the rate) and the shape parameter of the Erlang distribution governing transitions. The parameter means are given in days.

delay	rate parameter	mean	shape	source
incubation period	*ρ*	9.4	2	WHO Ebola response team [37]
infectious period	*γ*	7.8	3	data
admission	*κ*_t_	variable	2	data
ETC stay (survivors)	*θ*	13.0	10	data
unsafe burial	*α*	1	1	assumption

In order to track changes in transmission owing to behavioural or other changes in the community, we assumed that both the proportion seeking healthcare and the transmission rate could change over time; the extent and direction of change was estimated during the model fitting process. Healthcare seeking was modelled as a sigmoid function,
2.1
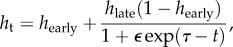
where *h*_early_ and *h*_late_ are the proportion of people seeking healthcare early and late in the epidemic, respectively, *τ* is an inflection point of the sigmoid and *ε* is the speed of change. This functional form ensured that healthcare seeking was either monotonically increasing or decreasing, as we would expect attitudes to healthcare to either improve or worsen during the course of the outbreak as a result of behavioural changes and health promotion activities, but not undergo rapid jumps.

The time-varying transmission rate *β*_t_, on the other hand, was assumed to be more likely to undergo sudden changes if there were, for example, superspreading events or spatial or other heterogeneities in transmission. We therefore modelled it as a weekly varying random variable that followed a normal distribution with given mean 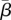 and standard deviation *σ*
2.2
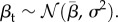


Assuming that no transmission occurs in an ETC or during a safe burial, the force of infection at any time is
2.3
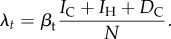
Assuming that there was no depletion of susceptibles (*S* ≈ *N*), the time-varying community reproduction number (i.e. the average number of infections caused by an infected person who does not seek healthcare) is
2.4
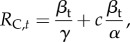
the two parts of the sum representing transmission before and after death. For those seeking healthcare and isolated (and, if necessary, safely buried), this changes to approximately:
2.5
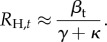


An exact calculation of this reproduction number calculated using a next-generation matrix [38] is given in the electronic supplementary material. It deviates slightly from the one in equation (2.5) because of the discrete nature of the multiple sequential compartments introduced to obtain Erlang-distributed transmission times.

The ratio between the two reproduction numbers is approximately
2.6
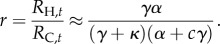


We calculated a range of values for this ratio using bootstrapped parameter values estimated from the line list of cases and using the exact formula for *R*_H_ given in the electronic supplementary material.

The overall reproduction number at any time is
2.7
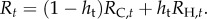
Incident ETC admissions were aggregated at a weekly level, and reporting assumed to follow a Poisson distribution centred around weekly incidence. The likelihood of each trajectory was obtained by multiplying the probability densities of observation at each time point, and the likelihood of a given set of parameter values was estimated using a particle filter [39]. The model was fitted to the time series of ETC admissions of confirmed and probable EVD cases using a Bayesian approach. Metropolis–Hastings Markov chain Monte Carlo in combination with the estimated likelihoods from the particle filter (a method called pMCMC) was used to sample parameter combinations and state trajectories from the posterior distribution of the parameters [40]. The filtered trajectories of the transmission rate *β*_t_ could then be used to track the behaviour of the transmission rate over time [31,41], and we used this to calculate the corresponding time-varying overall reproduction number (the average number of secondary cases generated by each case). Generally, if at any time the overall reproduction number is greater than 1, case numbers will typically rise whilst if it is less than 1, case numbers will generally decline.

The initial conditions and the parameters of the time-varying rates were estimated during the fitting process: the number of infectious people in Lofa County on 26 May 2014 (one week before the beginning of the time series of ETC admissions), the mean and standard deviation of the transmission rate, and the four parameters governing the proportion of the population seeking healthcare (*h*_early_, *h*_late_, *ε* and *τ*).

The stochastic elements of the model were updated at every week-long time step, and the model run as ordinary differential equations between time steps. The fitting procedure was implemented using *libbi* [42] with the *rbi* [43] and *rbi.helpers* [44] *R* packages on a high-performance computing cluster. After affirming that the method could recover the time-varying transmission rate from simulated data, we fitted the model using 100 parallel chains of 10 000 iterations each in the Markov chain Monte Carlo sampler. Convergence and mixing was confirmed visually. All code and data are available online at http://github.com/sbfnk/ebola_lofa, and instructions for reproducing the modelling results given in the electronic supplementary material.

### (c) Counterfactual scenarios

We adapted the model fitted to the ETC admissions data to explore counterfactual scenarios. To measure the impact of bed availability, we considered a model where limitations in capacity would prevent patients from getting admitted to the ETC. To do so, we modified our model to keep track of the number of people currently in the hospital. If this was at capacity, the infected individual would stay in the community and keep transmitting until there was space. After verifying that a model with estimated bed capacity as it was available during the outbreak (calculated as the maximal daily number of patients in the ETC up to any given week minus the current number of non-Ebola patients) yielded the observed epidemic, we explored hypothetical scenarios where there would be either (i) no expansion of bed capacity (and unlike the case in reality, rejection of patients once all beds were occupied) or (ii) no changes in healthcare-seeking behaviour, i.e. *h*_t_ fixed to its value in the first week.

## 3. Results

Between 18 March and 9 November 2014, a total of 466 confirmed and probable cases of EVD were identified (453 confirmed, 13 probable), of which 396 were ETC admissions (385 confirmed) and 70 community deaths (68 confirmed). The peak incidence of 67 reported new confirmed and probable cases of EVD occurred during the week of 18–24 August (58 patients admitted to the ETC, nine community deaths). This was followed by a steady decline to one confirmed case admitted to the ETC and no confirmed community death during the week of 20–26 October (figure 2). The number of community deaths reported (and thus provided a safe burial) increased from the end of June to a peak in the period from mid-September to mid-October, but these were progressively less likely to be owing to EVD. The proportion of community deaths confirmed positive remained near 90% until mid-August, then declined thereafter. Similarly, the proportion of ETC admissions that were confirmed EVD positive declined from 90% in mid-June to around 50% in mid-September. A detailed description of the outbreak and the response can be found in box 1.

Males (48%) and females (52%) were equally affected by the outbreak. Most (63%) of the cases were older adults (greater than or equal to 40 years of age), a third (33%) were young adults (15–39 years of age), whereas a minority were children (less than 15 years of age; 2%). Overall, 310 (67%) of the admitted patients with confirmed or probable EVD died of the disease. Within Lofa County, the case fatality rate did not differ by district of residence, between men and women, nor did it change significantly over time. Of the confirmed and probable cases of EVD, 21 (5%) were healthcare workers, of whom 14 (67%) died, six recovered and one had an unknown outcome.

**Figure 3.**
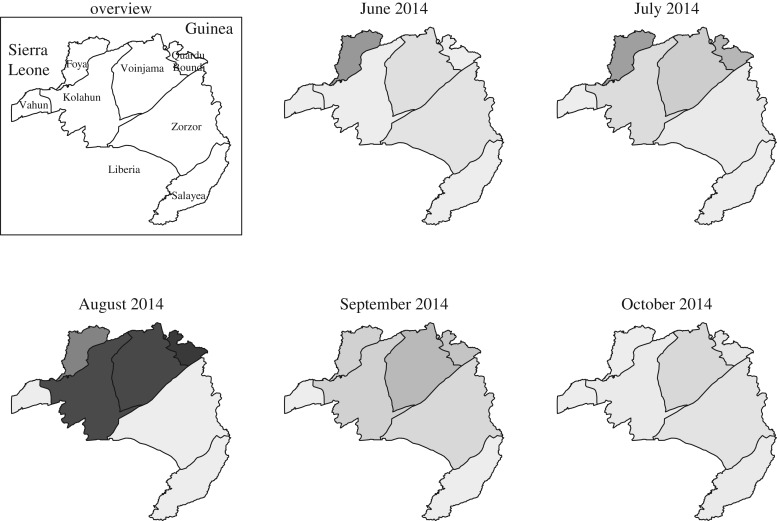
Geographical progression of the epidemic. Shades of grey indicate the number of Ebola cases admitted to the ETC, darker shades reflecting more cases (maximum: 59 in Quardu Bundi district, August).

The epidemic first affected the western districts of Foya, Kolahun and Vahun, and later the eastern districts of Voinjama, Zorzor and Salayea, Quardu Boundi (figure 3). No cases of local origin were seen in Foya, Kolahun or Vahun districts after mid-September, whereas local cases persisted in the eastern districts until the end of October. During the final weeks of October, more than half of the cases of EVD were exposed outside of Lofa County, in either Monrovia or Guinea.

The delay between symptom onset and ETC admission increased from a median of 3 days in June (50% of cases being admitted within 2–4 days), to 5 in July (50% between 3 and 7 days), before dropping again to 3 in August (50% between 2 and 5 days), September (50% between 2 and 5 days) and October (50% between 2 and 4 days). Weekly changes in the delay to admission can be seen in figure 2.

Box 1. **Outbreak and response.**

*The early outbreak*. The index case in Liberia, a 35 year old woman from Foya, had travelled to Guinea and became ill on 13 March. She was brought back to Foya, Liberia on 17 March by a 30 year old female relative and was admitted the following day to the Borma Hospital, Foya. She was confirmed EVD positive by testing at the Institut Pasteur Laboratory, Conakry, and died 20 March. Her female relative subsequently fell ill and was hospitalized at Foya Borma Hospital, but later fled to Monrovia continuing on to Firestone, Margibi County, Liberia where she died of confirmed EVD. Two male healthcare workers from the hospital, who had cared for the index case, aged 48 and 46, fell ill, were confirmed positive, and died on 31 March and 4 April, respectively. A 30 year old woman who had washed the clothes of the index case during her illness also fell ill, was confirmed positive and died on 1 April. Aside from this cluster, another case was confirmed in a 55 year old female from Foya who had returned from Guinea during the three weeks prior to the onset of her illness on 31 March 31. With the support of WHO, all deaths of confirmed cases received a safe burial. Following this cluster that occurred exclusively in Foya town, no further cases were confirmed in Lofa County until early June.

The second wave of the epidemic began in Lofa County on 6 June when a case was confirmed in a 46 year old man from Foya district who had been partially trained as a nurse. In May, he had travelled to a nearby village in Sierra Leone where he had cared for his ill mother, administering two intravenous infusions and two injections of medication. His mother died later that month from confirmed EVD. After returning to Liberia, he developed symptoms on 2 June, and was admitted to the ETC, Foya, on 5 June. He was confirmed positive and subsequently discharged recovered.

In the second and third weeks of June, a second large cluster of cases occurred in an extended family in Foya. The 65 year old matriarch of the family who lived with her husband in nearby Sierra Leone became ill on 20 May. She was taken to Foya by her family where she was admitted briefly to the Foya Borma Hospital with suspect EVD. As she was not improving, and before she could be confirmed as a case, she was taken to the family home in Foya where she began vomiting blood and died on 27 May. Her body was washed and dressed and transported in a rented minibus back to Sierra Leone where her burial took place. Subsequently, 16 people, including nine family members in Foya and seven individuals who attended the burial in Sierra Leone, died of confirmed EVD in both countries.

Incident cases stabilized until mid-July, but thereafter there was a rapid and geographically widespread increase in reported number of admissions to the Foya ETC and community deaths owing to confirmed EVD.

*National and international response*. The response to the EVD outbreak in Lofa County was led by the district and county-level MoH health team under the coordination of a National Ebola Task Force which was established on 30 March 2014 and comprised government agencies and international partners. On declaring the outbreak, the MoH recommended the avoidance of personal contact through handshakes, hugging and community gatherings and a halt to traditional burials, which typically involve family members washing the body and touching the corpse.

In early April, the WHO conducted a needs assessment, the US Centers for Disease Control and Prevention supported the development of an epidemiological surveillance and contact tracing system and, from its existing operational base in Guéckédou, Guinea, Médecins Sans Frontières (MSF) provided technical advice and training in community health promotion, case management and the establishment of isolation facilities, but was not involved in the direct provision of services in Lofa County until August.

By mid-July, WHO, MoH and MSF identified significant gaps in the response to the now-accelerating epidemic: low coverage of contact tracing, denial and resistance in communities, weak data management, inadequate infection prevention and control in health facilities, insufficient outreach capacity, insufficient safe burial capacity, and weak leadership and coordination at a national level [45]. WHO provided training for a cadre of General Community Health Volunteers and supervisors in an effort to improve the incomplete contact tracing. A comprehensive assessment of financial, logistical and human resource needs was incorporated into the Liberia Operational Plan for Accelerated Response to the Re-occurrence of Ebola Epidemic [46].

By early August, however, it was apparent that the epidemic in Lofa County and elsewhere in Liberia was spreading out of control. The national government declared a 90 day state of emergency on 6 August, imposing a 21.00 curfew, closing markets, schools and other public institutions and restricting both public gatherings and the movement of people across international borders. A number of communities restricted the entry of individuals from elsewhere, especially those coming from other districts and neighbouring countries. On 7 August, WHO declared the EVD epidemic in West Africa a Public Health Emergency of International Concern, invoking the 2005 International Health Regulations [47] and establishing an IHR committee for the EVD outbreak.

*Clinical interventions*. The first cases of EVD in Lofa County were identified and treated at Borma Hospital in Foya, an existing MoH facility. In response to the need for improved infection prevention and control, a 10 bed ETC was established on 20 April 2014 by the MoH with technical assistance from MSF, in an existing building located on the edge of the town of Foya that had formerly served as a refugee transit centre. Nursing, hygiene and administrative staff of the MoH were seconded to this unit with training provided by MSF. However, from the second week of April until the end of May, no further cases were identified in Lofa County. During this period, both the ETC and staff were on standby and community health promotion activities were minimal. Key informants indicate that most residents at the time doubted that Ebola was truly present in their communities.

With the reappearance of EVD in June, a non-governmental humanitarian organization, supported by MSF, took over the management of the Foya ETC. However, rapidly increasing numbers of EVD cases in July led to overcrowding in this facility with up to 40 patients, and necessitated the construction of a new 100 bed ETC of wood and plastic sheeting adjacent to the original centre in early August. Patients were transferred into the new facility, built and managed thereafter by MSF, on 10 August. The new ETC was designed to emphasize transparency in its activities by allowing family and community members to see patients receiving care. Provisions were made to house out-of-town patients' families and to allow visits at a safe 2 m distance.

Beginning in September, an MSF psycho-social team, comprising national and international mental health professionals, provided counselling and support for recovered ETC patients and their families both during their hospitalization and on their return to their home communities.

By July, many health structures were closed or operating only intermittently owing to fear of disease transmission on the part of both patients and staff. However, beginning at the end of August, the MoH and several international partners provided training and equipment to strengthen infection control and triage practice in the existing healthcare structures, salary supplements to Liberian staff engaged in the Ebola response, and vehicles and fuel to the MoH health teams [48].

*Community-based interventions*. A protocol to systematically investigate suspect cases of EVD in the community was established in early August, implemented first in the Foya and Voinjama districts, and later in the rest of the county. MSF and MoH set up Ebola telephone ‘hotlines’, enabling community members to alert the healthcare team to suspect cases and deaths. Calls were triaged, and an outreach team dispatched to investigate suspect cases or deaths. Transport of suspect cases, home decontamination and safe burial of corpses was performed according to established protocols [15]. The patient or, in the case of a death, the family of the deceased, would be interviewed using the national Ebola Case Notification Form to determine if the individual met the WHO definition of a suspect or probable case. If so, personal protective equipment (PPE) would be donned by the staff, the patient assisted into the ambulance (the cargo area of a pick-up truck covered with a canopy) for transport to the ETC, and the home of the patient decontaminated. If more than one patient was transported, all patients wore an impermeable gown, gloves and surgical mask. In the case of a suspect or probable death owing to EVD, a safe burial would be performed and the home of the deceased decontaminated according to established protocols [15,49]. From August, in addition to responding to hotline alerts, MSF outreach teams visited communities with ongoing transmission chains to conduct active case finding and bring suspect cases to the ETC for assessment.

Contacts of suspect cases and deaths were registered by the DHO at the time of case notification. Contact tracing was conducted by community volunteers supervised by MoH staff for 21 days following their last suspected exposure, either in person or by mobile phone, according to WHO protocols [50]. Accurate indicators of the completeness of contact tracing were not available to us; however, it was felt by key informants to have become progressively more complete from September onwards.

Health promotion efforts by several non-governmental organizations began in March, but were of limited geographical reach and intensity owing to limitations in transportation and human resources. In the run-up to the county election in July, at least one candidate distributed distinctive hand washing buckets to a number of larger villages as a preventive measure. Yet, in July, an outreach team was forced to withdraw from one community when it met with community opposition and violence. With an increased availability of vehicles and personnel from MSF and other international partners after mid-August, health promotion activity intensified and extended to outlying communities. MSF anthropologists and health promoters together with Liberian staff knowledgeable about local customs and languages, met first with local village leaders and later with community members in public meetings. Priority was given to those communities in which cases had recently occurred. The teams explored the community's beliefs about EVD, EVD prevention, the treatment and services provided at the ETC, and sought to clarify any misconceptions. Whenever possible, teams were accompanied on their visits by patients from the community who had been discharged recovered from the ETC. Representatives of each community, including community leaders, received training in EVD and were invited to tour the ETC. After July, there were no further reports of Ebola response teams encountering community resistance or violence.

Beginning in August, a range of communication strategies were used to promote the understanding of the real threat of Ebola, the early identification of symptoms, the prevention of spread of the disease, the use of the Ebola ‘hotline’ to refer suspect patients to the ETC, and the need for safe burial practices. Multiple, but not necessarily coordinated programmes, were launched by the national government, the United Nations Children's Emergency Fund (UNICEF), MSF and a number of media outlets. In September and October, radio and video clips using music, drama and personal testimonials in a range of local languages were made widely available. Local musicians appeared alongside MSF health promotion teams during community outreach. A number of MoH district health teams met with community leaders, youth groups, unions, marketing associations and community-based organizations to promote these messages.

**Figure 4.**
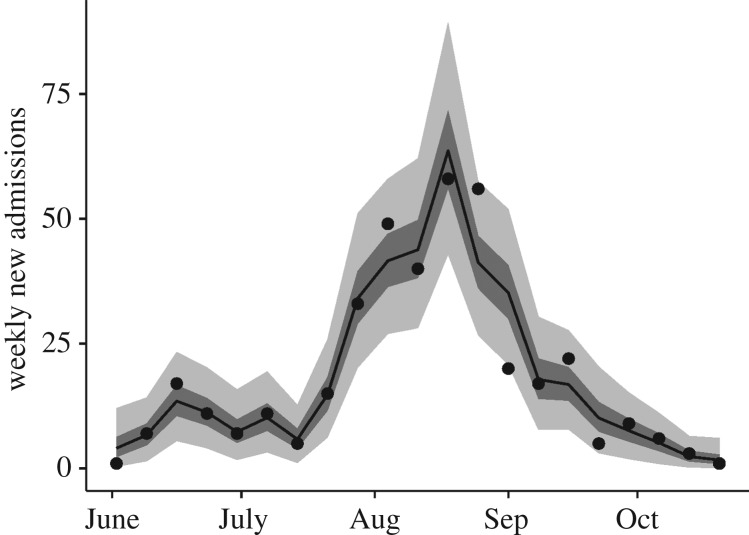
Model and data. Model fits to hospital admissions. Median: black line, IQR: dark shaded area, 95% credible interval: light shaded area. Data on weekly ETC admissions are shown as points. The shown fits are filtered trajectories from the posterior distribution of simulated observations.

Our model reproduced the trend in hospital admissions between June and November (figure 4). Because of correlations between the parameters, they were relatively poorly identified: the mean estimate for the initial number of infected people was 34 (interquartile range, IQR, 9–43, 95% credible interval, CI, 3–146, uniform prior range 0–200) and was negatively correlated with the average transmission rate. The mean estimate of the mean community reproduction number was 1.5 (IQR 0.7–2.0, 95% CI 0.1–3.9, uniform prior range 0–10), itself positively correlated with the early proportion of people seeking healthcare. That is, for more people seeking healthcare a higher transmission rate was required to create the observed data, as a larger proportion of the population was being isolated. The mean estimate for the weekly standard deviation in the transmission rate (on the scale of the community reproduction number *R*_C_) was 4.5 (IQR 3.1–5.6, 95% CI 1.8–9.0, uniform prior range 0–10).

The estimated community reproduction number (i.e. the average number of new cases caused by each case if there were no beds available to isolate them) shows a pattern of multiple sub-outbreaks followed by brief periods of no transmission, combined with an overall decline (figure 2*f*). The longest sustained period of transmission was in the five weeks between 30 June and 4 August, during which the reproduction number can be seen to steadily increase. The subsequent peaks in mid-August and early and late October are significantly smaller. After the first week of October, more than 78% of posterior samples of the reproduction number were below 1.

We estimated that healthcare-seeking behaviour increased during the course of the outbreak, from a median estimate of 36% (IQR 16–67, 95% CI 3–96) of the population seeking healthcare at the beginning of June to a median estimate of 87% (IQR 72–96, 95% CI 26–100) at the end of October. By comparing the late to early healthcare-seeking behaviour on a sample-by-sample basis, our median estimate suggests that individuals were 2.0 (IQR 1.3–4.3, 95% CI 1.0–21) times as likely to seek healthcare late in the epidemic as early. With the parameters in our model, isolation in the ETC reduced the reproduction number of those that seek healthcare to a fraction *r* = 0.38 (median estimate, IQR 0.37–0.39, 95% CI 0.37–0.40), or by 66%. If individuals were assumed to be twice as infectious during the later stage of infection and burial, the effect was even stronger, with *r* = 0.33 (median estimate, IQR 0.32–0.34, 95% CI 0.31–0.35) or a 67% reduction of the reproduction number of those seeking healthcare (implying safe burial).

**Figure 5.**
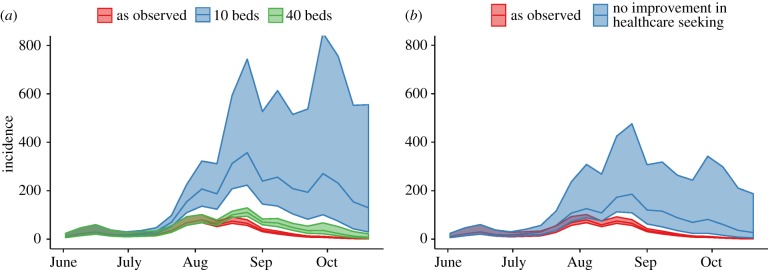
Incidence of EVD under alternative scenarios. Incidence under the alternative scenarios of (*a*) lower ETC capacity and (*b*) no improvements in healthcare-seeking behaviour. Shown are the median (line) and IQR (shaded area) at each time point.

Aggregated simulated trajectories of total incidence (in the community and the ETC) under the scenarios of (i) no bed expansion (and rejection of patients if no beds were available) and (ii) no change in healthcare-seeking behaviour from early in the outbreak are shown in figure 5. The exact number of cases and deaths prevented depended on the exact proportion of healthcare seeking, which was only weakly identified in our model. Comparing the scenarios on a (posterior) sample-by-sample basis, we found that keeping capacity at 10 beds (the official capacity) increased the overall number of cases by a factor of 4.7 (median, IQR 2.1–14, 95% CI 1.1–59), while keeping it at 40 (the number of patients for which the 10 bed ETC was used during the period of overcrowding in July to early August) increased the number of cases by a factor of 1.4 (median, IQR 1.2–1.8, 95% CI 1.0–2.8). Keeping healthcare-seeking behaviour as it was at the beginning of the outbreak increased the number of cases by a factor of 1.9 (median, IQR 1.2–5.2, 95% CI 1.0–109).

## 4. Discussion

Lofa County was the first region in Liberia to experience the 2013–2015 EVD outbreak in West Africa, and it was struck severely. A rapid rise in cases over the summer of 2014 was followed by a fall in cases towards the end of August 2014, and control by the end of October. The behaviour of the epidemic in Lofa mirrors what has been observed across many regions of West Africa, and a better understanding of this behaviour can yield important lessons for investigating the epidemic elsewhere. Here, we analysed the impact of different clinical and community-based interventions that were put in place at different times, in an attempt to pinpoint what exactly turned the epidemic around.

The main clinical intervention was the expansion of the ETC in Foya from a capacity of 10 (although in fact used for up to 40 patients during periods of overcrowding) to 100. We have estimated that the provision of isolation enabled through the expanded ETC reduced the reproduction number of those who entered the ETC by about two-thirds and thus made a strong contribution to the substantial decline in case numbers observed in the three weeks following the opening of the expanded ETC. Without the expansion, the outbreak would have continued to grow for weeks longer, and we estimated that hundreds of lives were potentially saved by this measure.

The national government imposed a state of emergency in Liberia on 6 August. By closing markets, schools and restricting public gatherings and movement across international borders, this measure may have altered patterns of contact in communities and contributed to preventing geographical spread, but possibly not to a reduction in disease transmission, much of which occurs among family members [51]. At the same time, the delay between the development of symptoms and hospitalization of cases increased when case numbers were growing rapidly before stabilizing after the ETC was expanded, and declining further as the outbreak was well under control. Key observers have suggested that the increase observed in June and early July was an indicator of prevailing community attitudes at the time: denial of the EVD outbreak, and reluctance to present for care because of the stigma associated with the disease. With the ETC operating at capacity, this could explain why it took longer to admit patients. More prompt presentation for care from August onwards may reflect a combination of greater availability of ETC beds, more positive attitudes toward treatment, and greater community awareness of the disease, however, one cannot ascertain the relative contribution of these factors.

From the modelling analysis, it appeared that the expansion of the ETC capacity, occasional interruptions of transmission and improvements in healthcare-seeking behaviour all contributed to mitigating the epidemic and eventually stopping it. Two features stood out in the time-varying behaviour of the transmission rate: one is that the epidemic in Lofa appeared to have consisted of several subepidemics which intermittently stopped before starting again. This is consistent with geographically heterogeneous dynamics where outbreaks flare up and are subsequently controlled by a combination of local behavioural change and targeted intervention, but potentially trigger outbreaks elsewhere. The second feature is the overall decline in the reproduction number from August, leading to eventual extinction. This decline has been previously observed using a model of behavioural change that did not include the detailed information of the outbreak which we included here [24]. It is impossible to establish with certainty the reasons for the decline from the available data, but a strong role of concurrent efforts of the provision of clinical care and isolation and community engagement is consistent with our findings.

The exact extent of healthcare-seeking behaviour was difficult to establish from fitting to hospital admission data alone, which did not contain any information on how many cases may still have been occurring in the community but went unreported. Still, our model suggests that it did appear to improve between the beginning and the end of the epidemic. Interestingly, much of the increase in healthcare-seeking behaviour appeared to happen before the start of health-promotion activities, indicating that attitudes may have changed substantially before the onset of these activities. In this context, it is important to bear in mind that the time course of healthcare-seeking behaviour was constrained by the particular structure of the model we chose. While the transmission rate was allowed to vary randomly and therefore increase and decrease at any point in time, we assumed that healthcare-seeking behaviour could only change in one direction over the course of the outbreak. If we had allowed similar flexibility in the way we modelled healthcare-seeking behaviour to the way we modelled the changes in the transmission rate, it might not have been possible to pinpoint these changes. The behaviour we found was consistent with observations that overall attitudes towards health institutions improved during the course of the epidemic. It helps explain the very sharp rise of cases admitted to the ETC in July (which was stronger than could be explained with a constant transmission rate), and it aligns with the fact that the outbreak was eventually ended relatively rapidly. Without further information on community behaviour, however, it remains difficult to establish these relationships with certainty.

More generally, it would be desirable in the course of future outbreaks to implement concurrent community surveillance to directly monitor total deaths and their causes, and measure a limited number of key social variables. Assessing all-cause mortality within the community could provide an estimate of the number of suspect EVD cases in the community that did not present to the ETC for care or were not provided a safe community burial. This would allow ongoing measurement of healthcare- and safe-burial-seeking behaviour, including an estimate of the proportion of EVD in the community not being identified and properly managed. Similarly, if one had direct information on public attitudes (e.g. perception of the ETC, EVD and its transmission, willingness to seek healthcare if ill), such information could assist in not only more accurately understanding and modelling the role of these factors in the evolution of the outbreak, but also in tailoring social mobilization efforts to increase their effectiveness in particular communities. While such studies are clearly secondary to immediate efforts to mitigate transmission and care for the sick, they could provide crucial information for evaluating and tailoring the ongoing outbreak response and planning for future such events.

## Supplementary Material

Modelling results

## Data accessibility

All data are available at http://github.com/sbfnk/ebola_lofa.
