# Supplementary material for: The impact of control strategies and behavioural changes on the elimination of Ebola from Lofa County, Liberia
Source: Philos Trans R Soc Lond B Biol Sci. 2017 Apr 10;372(1721):20160302. doi: 10.1098/rstb.2016.0302 (PMC5394640; doi:10.1098/rstb.2016.0302)
Supplement: Modelling results [file rstb20160302supp1.pdf]

Electronic supplementary material: Modelling results

The impact of control strategies and behavioural changes on the elimination of Ebola from Lofa County, Liberia

Sebastian Funk, Iza Ciglenecki, Amanda Tiffany, Etienne Gignoux, Anton Camacho, Rosalind M. Eggo, Adam J. Kucharski, W. John Edmunds, Josephus Bolongei, Phillip Azuma, Peter Clement, Alpha Tamba, Esther Sterk, Barbara Telfer, Gregory Engel, Lucy Anne Parker, Motoi Suzuki, Nico Heijenberg, Bruce Reeder

Introduction

This document contains the R code necessary to reproduce the results in "Epidemiology, events and control strategies towards eliminating of Ebola from Lofa County, Liberia." All code and data used here are in a github repository.

Required packages

```
cran_packages <- c("devtools", "dplyr", "tidyr", "cowplot", "stringi", "truncnorm")
github_packages <- c("libbi/RBi", "sbfnk/RBi.helpers")
```

```
for (package in cran_packages) {
    install.packages(package)
}

library("devtools")

for (package in github_packages) {
    install_github(package)
}
```

Generate MCMC chains

Generating MCMC chains requires a working install of libbi v1.2.0.

Edit the lines starting `code_dir <-` and `output_dir <-` in the script 'sample_posterior.r' from the R/ directory in the github repository to point to the correct directories, and run it using

```
Rscript sample_posterior.r -n 10000 -p 1000 -r independent -t $NUM_THREADS -i 10 -q -l -f \
                           -o ebola_lofa_$NB -c 16384 -b $NB
```

where you should set `$NUM_THREADS` to the number of concurrent threads to run (i.e., the number of CPU cores available) and `$NB` to a running number (1,2,3,. . .) for each chain to generate (100 in the manuscript).

Analyse MCMC chains

Set the `output_dir` to the value used in `sample.posterior.r`

```r
output_dir <- "insert your directory here"
```

Load required packages

```r
for (package in c(cran_packages, github_packages)) {
    library(sub("^.*/", "", package), character.only = TRUE)
}
```

Get MCMC chains

```r
mcmc_files <- list.files(output_dir, pattern = "ebola_lofa_[0-9]+\\.rds", full.names = TRUE)

res <- list()

model <- NULL
obs <- NULL
input <- NULL
data <- NULL

for (file in mcmc_files) {
    res[[file]] <- readRDS(paste0(file))
    if (is.null(model))
        model <- bi_model(sub("\\.rds$", ".bi", file))
    if (is.null(obs))
        obs <- readRDS(sub("\\.rds$", "_obs.rds", file))
    if (is.null(input))
        input <- readRDS(sub("\\.rds$", "_input.rds", file))
    if (is.null(data))
        data <- readRDS(sub("\\.rds$", "_data.rds", file))
}
```

Combine into one chain

```r
np_translate <- NULL
combined <- lapply(names(res[[1]]), function(x) {
    z <- rbindlist(lapply(seq_along(res), function(y) {
        data.table(res[[y]][[x]])[, `:=`(unique_np, paste(np, y, sep = "_"))]
    }))
    if (is.null(np_translate)) {
        np_translate <- data.table(unique_np = unique(z$unique_np), new_np = seq_along(unique(z$unique_[illegible]
            1)
    }
    z <- merge(z, np_translate, by = "unique_np")
    z[, `:=`(np, NULL)]
    z[, `:=`(unique_np, NULL)]
    setnames(z, "new_np", "np")
    setkey(z, np)
    z
})
names(combined) <- names(res[[1]])
```

Save

```r
saveRDS(list(traces = combined, input = input, obs = obs, data = data), paste0(output_dir,
    "/lofa_combined_traces.rds"))
```

Sample observations

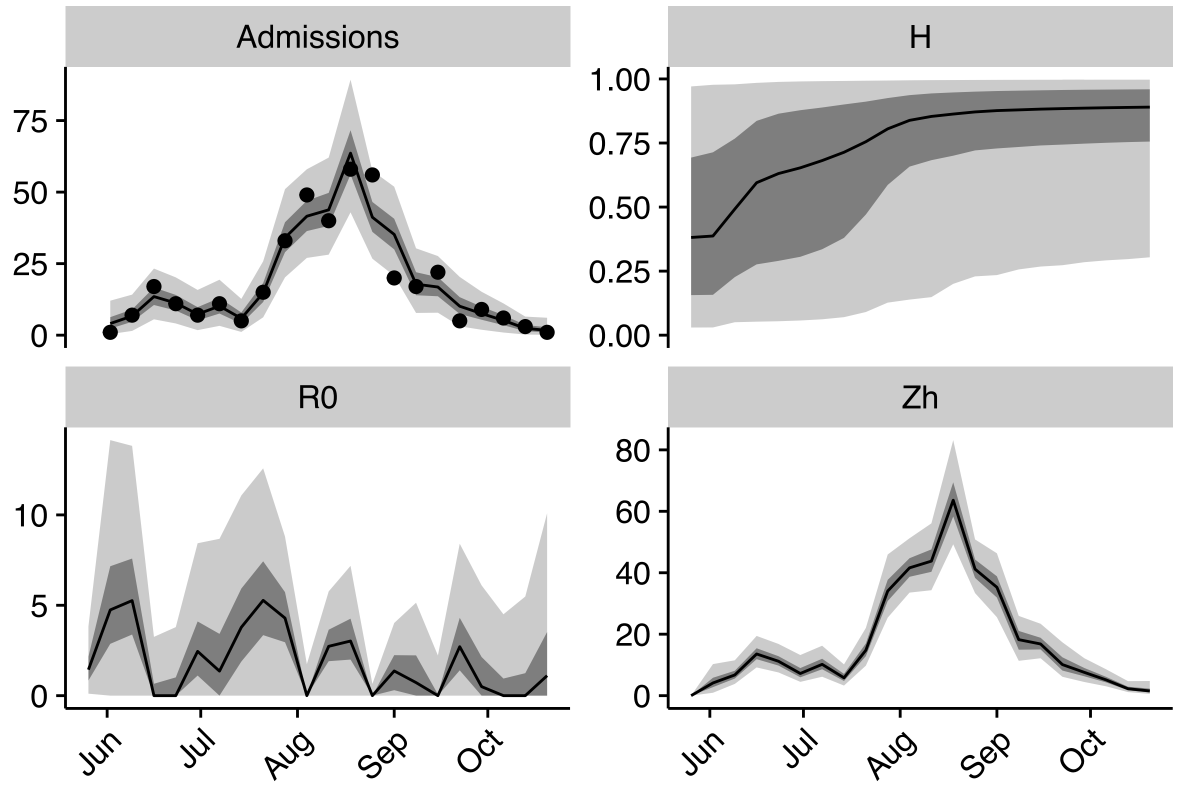


```
combined$Admissions <- copy(combined$Zh)
combined$Admissions[, `:=`(mean, value)]
combined$Admissions[, `:=`(sd, sqrt(value))]
combined$Admissions[, `:=`(value, rtruncnorm(n = .N, a = 0, mean = mean, sd = sd))]
combined$Admissions[is.na(value), `:=`(value, 0)]
```

Plot

```
p <- plot_libbi(combined, model, data = data, date.origin = as.Date("2014-06-02") -
    7, date.unit = "week", data.colour = "black", densities = "histogram", plot = FALSE)
```

State estimates

```
p$states
```

Figure 3:

```
p_obs <- plot_libbi(combined, model, data = data, date.origin = as.Date("2014-06-02") -
    7, date.unit = "week", data.colour = "black", densities = "histogram", plot = FALSE,
    states = "Admissions")
p_obs$states
```

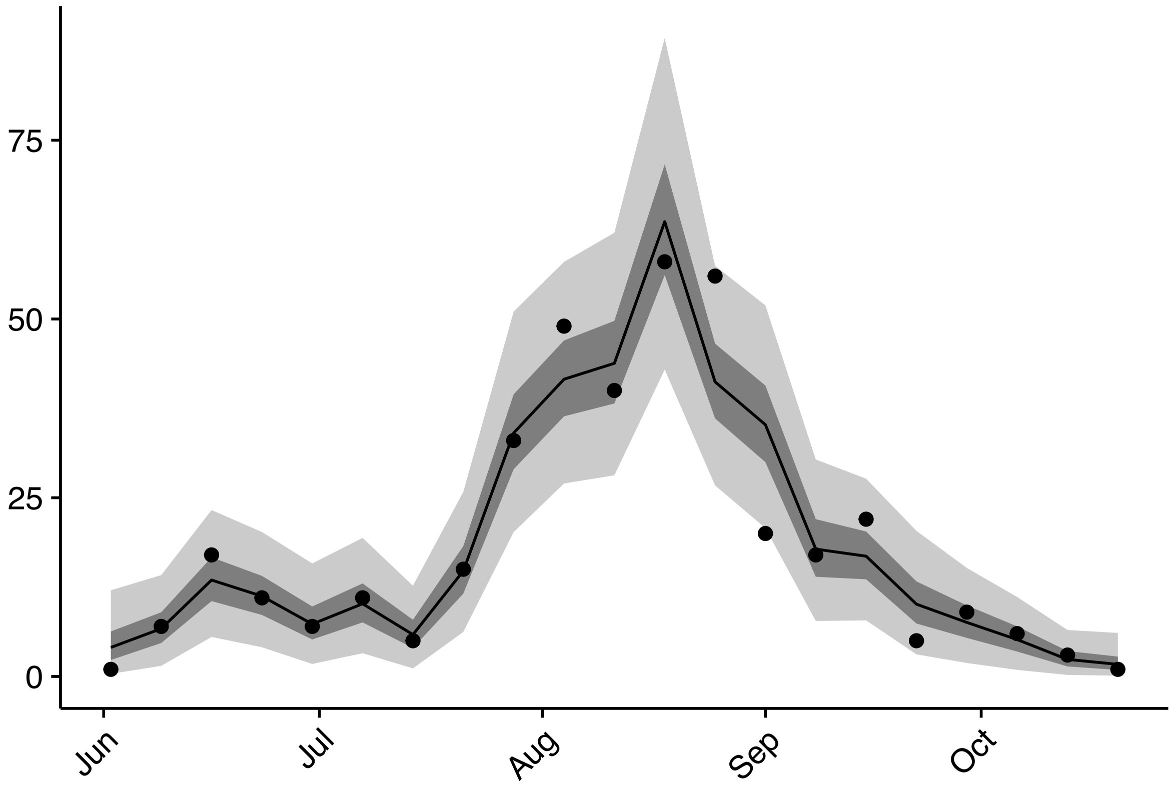

Traces

```
p$traces
```

Densities

```
p$densities
```

Analyse

Initial number of infected

```
combined$p_Inf[, list(mean = mean(value), min.50 = quantile(value, 0.25), max.50 = quantile(value,
    0.75), min.95 = quantile(value, 0.025), min.95 = quantile(value, 0.975))]
```

```
##        mean  min.50   max.50   min.95   min.95
## 1: 34.71357 9.30621 42.62314 3.376042 159.4193
```

Mean reproduction number

```
combined$p_R0[, list(mean = mean(value), min.50 = quantile(value, 0.25), max.50 = quantile(value,
    0.75), min.95 = quantile(value, 0.025), min.95 = quantile(value, 0.975))]
```

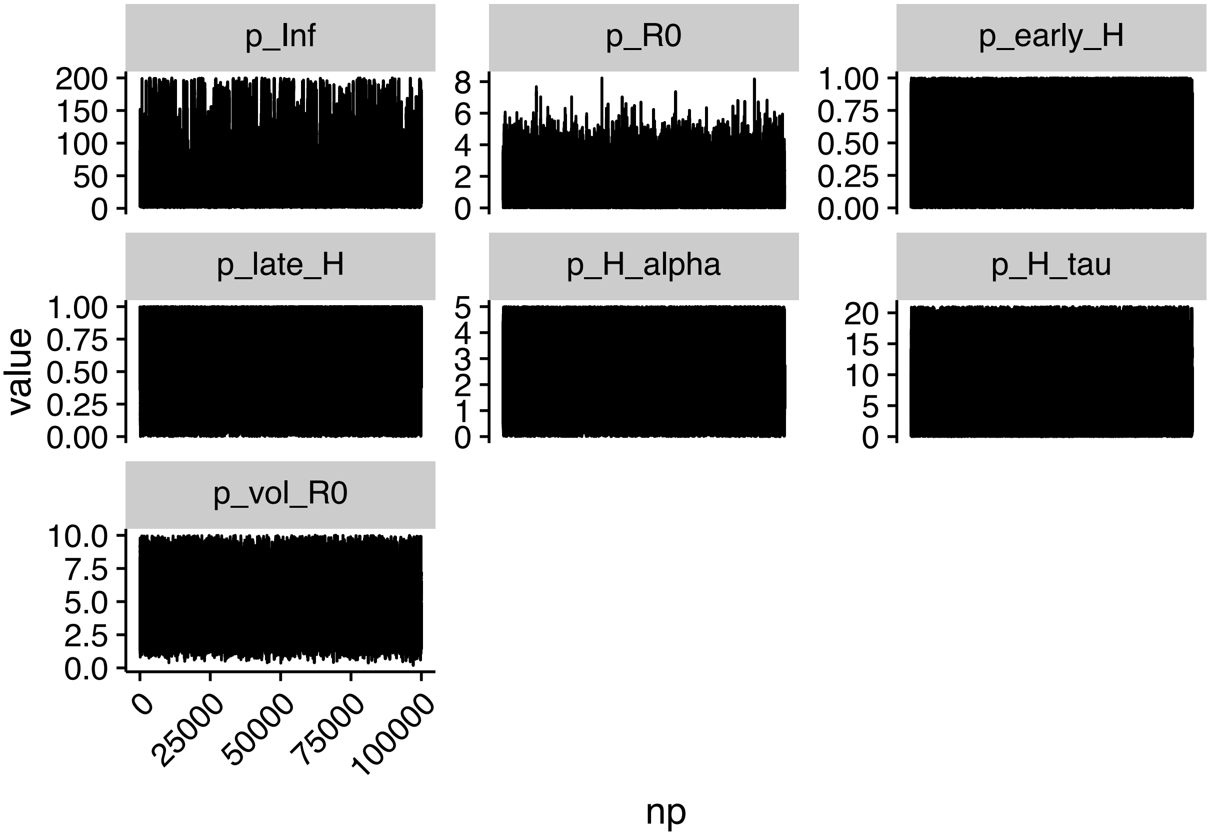
p_Inf
p_R0
p_early_H
p_late_H
p_H_alpha
p_H_tau
p_vol_R0
value
np
0
25000
50000
75000
100000

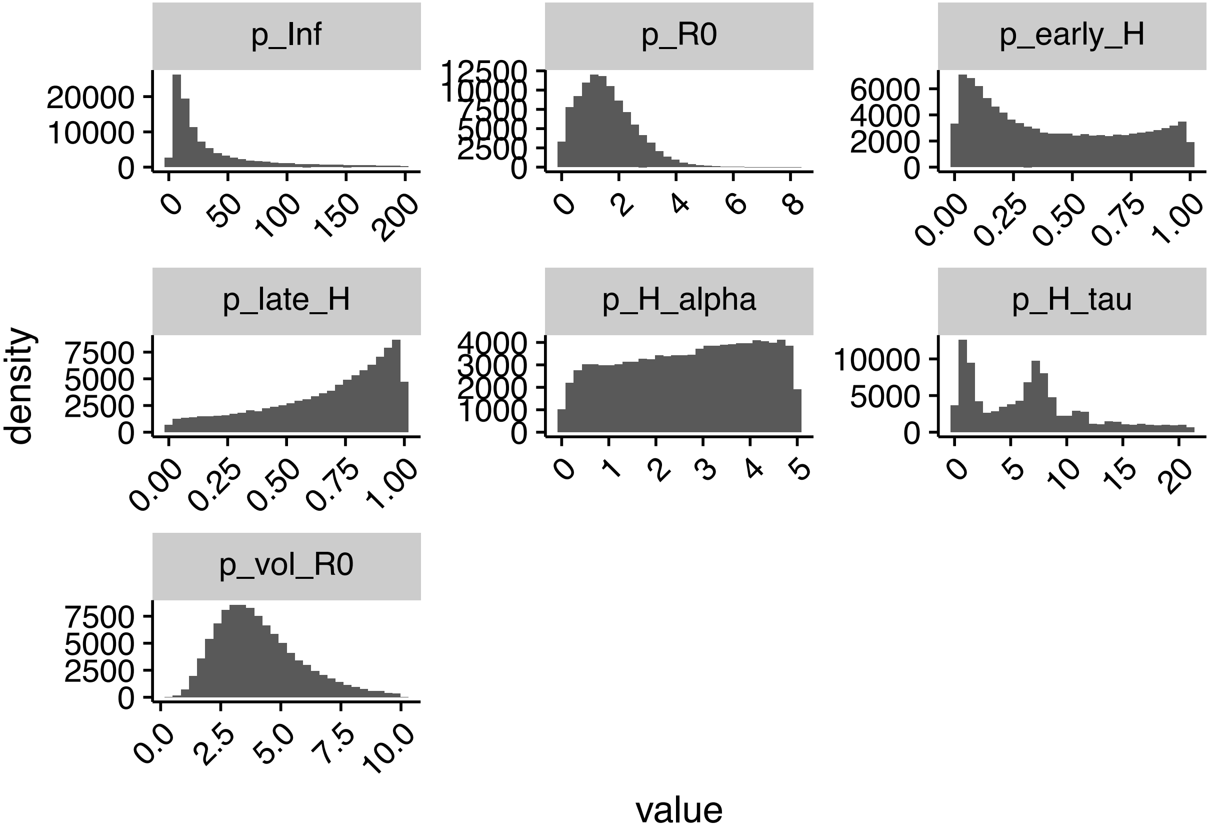
p_Inf
p_R0
p_early_H
p_late_H
p_H_alpha
p_H_tau
p_vol_R0
density
value

```
##        mean    min.50   max.50    min.95   min.95
## 1: 1.572017 0.8352451 2.158922 0.1078207 3.836637
```

Standard deviation of reproduction number

```
combined$p_vol_R0[, list(mean = mean(value), min.50 = quantile(value, 0.25),
    max.50 = quantile(value, 0.75), min.95 = quantile(value, 0.025), min.95 = quantile(value,
        0.975))]
```

```
##        mean   min.50   max.50  min.95   min.95
## 1: 4.095616 2.801514 5.090164 1.47265 8.352336
```

Early proportion seeking healthcare

```
elH <- combined$H[, list(early = value[1], late = value[length(value)]), by = np]
elH[, list(median = median(early), min.50 = quantile(early, 0.25), max.50 = quantile(early,
    0.75), min.95 = quantile(early, 0.025), max.95 = quantile(early, 0.975))]
```

```
##       median    min.50    max.50     min.95    max.95
## 1: 0.3813748 0.1563478 0.6930892 0.02990701 0.9707741
```

Late proportion seeking healthcare

```
elH[, list(median = median(late), min.50 = quantile(late, 0.25), max.50 = quantile(late,
    0.75), min.95 = quantile(late, 0.025), max.95 = quantile(late, 0.975))]
```

```
##       median    min.50    max.50    min.95    max.95
## 1: 0.8900784 0.7556231 0.9596605 0.3038647 0.9972571
```

Ratio between proportion seeking healthcare at last and first data point

```
elH <- combined$H[, list(early = value[1], late = value[length(value)]), by = np]
elH[, `:=`(ratio, late/early)]
elH[, list(median = median(ratio), min.50 = quantile(ratio, 0.25), max.50 = quantile(ratio,
    0.75), min.95 = quantile(ratio, 0.025), max.95 = quantile(ratio, 0.975))]
```

```
##      median   min.50   max.50   min.95   max.95
## 1: 2.015291 1.240955 4.616579 1.007414 24.29852
```
